# Accuracy of smartphone-based hearing screening tests: a systematic review

**DOI:** 10.1590/2317-1782/20212020380

**Published:** 2022-02-23

**Authors:** Inara Maria Monteiro Melo, Aline Roberta Xavier Silva, Rodolpho Camargo, Hannalice Gottschalk Cavalcanti, Deborah Viviane Ferrari, Karinna Veríssimo Meira Taveira, Sheila Andreoli Balen

**Affiliations:** 1 Universidade Federal do Rio Grande do Norte – UFRN, Natal (RN), Brasil.; 2 Faculdade de Odontologia de Bauru, Universidade de São Paulo – FOB/USP, Bauru (SP), Brasil.; 3 Universidade Federal da Paraíba – UFPB, João Pessoa (PB), Brasil.

**Keywords:** Audiology, Hearing Loss, Smartphone, Hearing Test, Mass Screening, Public Health, Audiologia, Perda Auditiva, Smartphones, Testes Auditivos, Programas de Rastreamento, Saúde Pública

## Abstract

**Purpose:**

To verify the accuracy of smartphone apps to identify hearing loss.

**Research strategies:**

A systematic review followed the PRISMA-DATA checklist. The search strategies were applied across four databases (Lilacs, PubMed, Scopus and Web of Science) and grey literature (Google Scholar, OpenGrey, and ProQuest Dissertations and Thesis).

**Selection criteria:**

The acronym PIRD was used in review. This included populations of any gender and all age groups. The Index test is the smartphone-based hearing screening test; the Reference test is the pure-tone audiometry, which is considered the gold reference for hearing diagnostics; the diagnosis was performed via validity data (sensitivity and specificity) to identify hearing loss and diagnostic studies.

**Data analysis:**

Two reviewers selected the studies in a two-step process. The risk of bias was assessed according to the criteria of the QUADAS-2.

**Results:**

Of 1395 articles, 104 articles were eligible for full-text reading and 17 were included. Only four met all criteria for methodological quality. All of the included studies were published in English between 2015 and 2020. The applications Digits-in noise Test (5 articles), uHear (4 articles), HearScreen (2 articles), hearTest (2 articles) and Hearing Test (2 articles) were the most studied. All this application showed sensitivity and specificity values between 75 and 100%. The other applications were EarScale, uHearing Test, Free field hearing (FFH) and Free Hearing Test.

**Conclusion:**

uHear, Digit-in-Noise Test, HearTest and HearScreen have shown significant values of sensitivity and specificity and can be considered as the most accurate methods for screening of hearing impairment.

## RESUMO

**Objetivo:**

Verificar a acurácia dos aplicativos de *smartphone* para identificar a perda auditiva.

**Estratégias de pesquisa:**

Uma revisão sistemática seguiu o *checklist* PRISMA-DATA. As estratégias de busca foram aplicadas nos bancos de dados Lilacs, PubMed, Scopus e Web of Science e na literatura cinzenta (Google Scholar, OpenGrey e ProQuest Dissertations and Thesis).

**Critérios de seleção:**

O anacrônimo PIRD foi usado na revisão. Incluiu populações de qualquer gênero e todas as faixas etárias. O teste Index foi o de triagem auditiva baseado em *smartphone*; o teste de referência foi a audiometria tonal; o diagnóstico foi realizado por meio de dados de validade (sensibilidade e especificidade) para identificação da perda auditiva e estudos diagnósticos.

**Análise de dados:**

Dois revisores selecionaram os estudos em um processo de duas etapas. O risco de viés foi avaliado de acordo com os critérios do QUADAS-2.

**Resultados:**

De 1395 artigos, 104 artigos foram elegíveis para leitura de texto completo e 17 foram incluídos. Apenas quatro preencheram todos os critérios de qualidade metodológica. Todos os estudos incluídos foram publicados em inglês entre 2015 e 2020. Os aplicativos mais estudados foram: *Digits-in-noise* (5 artigos), *uHear* (4 artigos), *HearScreen* (2 artigos), *hearTest* (2 artigos) e *Hearing Test* (2 artigos). Todos apresentaram valores de sensibilidade e especificidade entre 75 e 100%. Os outros aplicativos foram *EarScale*, *uHearing, Free Field Hearing* e teste *Free Hearing*.

**Conclusão:**

uHear, Digit-in-Noise Test, HearTest e HearScreen apresentaram valores significativos de sensibilidade e especificidade e podem ser considerados os métodos mais precisos para rastreamento de deficiência auditiva.

## INTRODUCTION

It is estimated that 460 million people live with hearing impairment worldwide. Of these, 40.19 million are in Latin America and the Caribbean, with a projection of 87 million people in 2050^(1)^. There is also an estimation that 1.1 billion young people (aged 12-35 years) are at risk of hearing loss due to exposure to noise in recreational environments^(2)^. Along with aging, this issue subsidizes part of the projections to increase the prevalence of hearing loss.

Hearing impairment has functional, psychosocial, and economic impacts at different life stages, especially if not identified and treated. In children, these impacts are more evident in language development and their learning processes. In adults, it can severely limit work capacity, and in the elderly, it can generate psychosocial impacts that can worsen aging and social isolation.

One of the main aspects of hearing loss health care is the prevention and identification of hearing loss using validated and accurate hearing screening instruments.

In the past few years, many methods for hearing screening have been developed in different countries, allowing individuals to perform the test with portable audiometric screening platforms with specialised professionals^(3)^, access to hearing health services in remote^(4-6)^ and/or applied automated hearing screening test with smarthphone or tablet at home without any specialised professionals^(7)^.

Using smartphones as a resource for hearing screening has been broadly studied since there are approximately 5.1 billion smartphones worldwide, in both urban and rural areas, in addition to the increasing availability of online services. That makes hearing healthcare even more accessible to end-users and promotes the so-called mHealth, which is the use of information and communication technologies to provide and improve healthcare services^(8-10)^.

There are currently thousands of health-related apps, which are relatively new to the mobile health scene and assess hearing loss by using smartphone hardware and earphones. Some of these apps accurately measure auditory thresholds, such as uHear, EarTrumpet, and hearScreen. Their authors argue that these methods are key in environments with limited resources, where high-end audiometry equipment is not available^(11,12)^.

To be considered an accurate instrument, hearing screening tests must be quick, simple, low-cost and have high sensibility and specificity. Other features such as self-administered, automated and use signals and noise equivalent to daily-life situations can optimize the use of these tests in hearing screening^(13-16)^.

Therefore, it is crucial to evaluate existing smartphone app-based hearing screening methods and discover whether they are indeed accurate, that is, if they measure the proportion of actual positives that are correctly identified as having a hearing loss as well as the proportion of actual negatives that are correctly identified as not having any hearing loss.

## PURPOSE

This systematic review aims to verify the accuracy of smartphone apps to identify hearing loss.

## RESEARCH STRATEGY

### Protocol and registration

A systematic review protocol based on the *Preferred Reporting Items for Systematic reviews and Meta-Analyses of Diagnostic Test Accuracy Studies* (PRISMA-DTA)^(17)^ was prepared and registered on the Prospective Register of Systematic Reviews (PROSPERO) under registration No. CRD42019126378.

### Search information

The question of this systematic review was “What is the accuracy of smartphone-based hearing screening tests for identifying hearing loss”?

Electronic search strategies were developed for each of the following databases: Latin American and Caribbean Health Sciences (LILACS), PUBMED (including MedLine), SCOPUS, and Web of Science. The authors performed an additional search in the grey literature, including Google Scholar, OpenGrey and ProQuest Dissertations and Thesis as well as a manual search in reference lists of the included studies following the recommendations of Greenhalgh and Peacock^(18)^. It embraced studies from all languages, no filter is applied as to the language of the articles and no restriction regarding age, sex and nor time of publication. The search strategies are available in Appendix 1. Experts were consulted to indicate additional studies that could be included. Reference management program Mendeley Desktop 1.19.2 was used for selecting references and removing duplicate articles. A free, online, collaborative systematic review app, Ryyan.qcri (Rayyan, Qatar Computing Research Institute)^(19)^, was used to read titles and abstracts. Search date on all databases and grey literature was July 10^th^, 2018, the search was updated on December 2^nd^, 2019 and July 20^nd^, 2020.

## SELECTION CRITERIA

### Eligibility criteria

The acronym PIRD is recommended for structuring the inclusion criteria that focuses on diagnosis study reviews. *P* stands for population, *I* for index test, *R* for reference test, and *D* for diagnosis of interest. This review included populations of any gender and all age groups. The Index test is the smartphone-based hearing screening test; the Reference test is the pure-tone audiometry, which is considered the gold reference for hearing diagnostics; the diagnosis was performed via validity data (sensitivity and specificity) to identify hearing loss and diagnostic studies^(20)^.

### Inclusion criteria

The authors included studies that relied on smartphone-based hearing screening tests to identify hearing loss to any degree and then compared their results to pure-tone audiometry, which is considered the reference standard for audiological evaluation.

### Exclusion criteria

The authors excluded studies that met the following criteria: 1. Studies that did not use phone apps; 2. Studies that were not audiology-related; 3. Studies that did not compare screening methods of phone apps with the reference standard (audiometry); 4. Studies that did not show any validity measurements (sensitivity and specificity) or did not show sufficient data to calculate them; 5. Comments, letters, conference, summary, personal opinions, clinical trials, case-control and cohort studies; 6. Unavailable studies.

## DATA ANALYSIS

### Study selection and data collection process

Two independent reviewers evaluated and selected the articles to be included. In phase one, both reviewers read the titles and abstracts independently and applied the eligibility criteria. In phase two, the same two reviewers read the full text. Any disagreements between the two reviewers that persisted after applying the eligibility criteria were resolved through consensus with a third reviewer. The final selection was based on the reading of the full texts when each of the following items was identified: author, year and country, sample and age group, app/test type, test procedures, sensitivity, and specificity.

### Risk of bias and applicability

Two independent authors performed an article quality assessment based on the Quality Assessment of Diagnostic Accuracy Studies (QUADAS-2)^(21)^. They assessed the risk of bias and applicability concerns in four main domains ('patient selection', 'index test', 'reference standard', and 'flow and time') and classified them as 'low', 'medium', or 'high'. Based on that information, the authors then used the Cochrane Collaboration's program Review Manager 5.3 to generate the figures.

### Summary measures

The information collected from the studies was quantitative – sensitivity and specificity values, negative and positive predictive values, prevalence and accuracy – and qualitative – sample size, age group, test procedure analysis, pass-fail criterion, and type of stimulus used in the test. That is due to the fact that the answer to this review's problem requires a detailed analysis of the studies for the accuracy evidence of smartphone app-based hearing screenings.

## RESULTS

### Selection of studies

**Figure 1:**
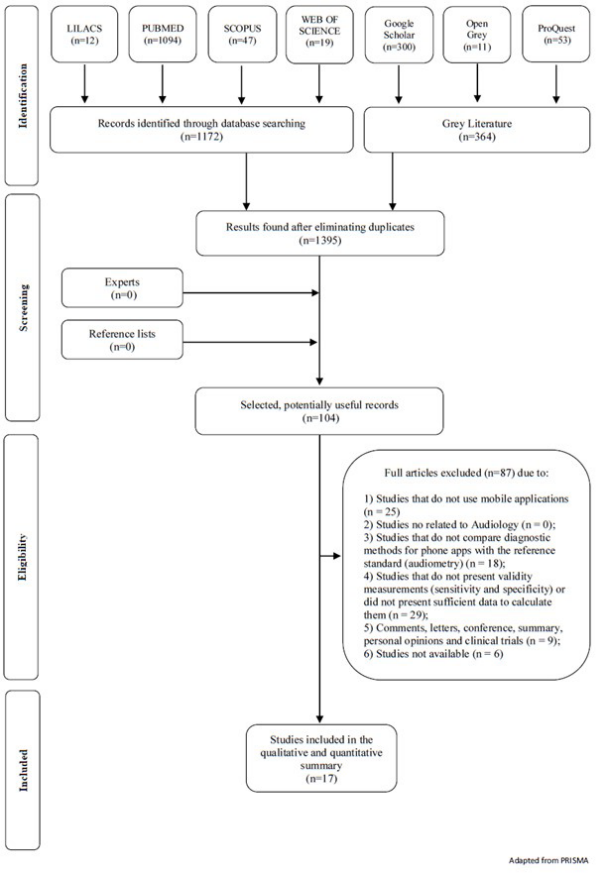
Flow Diagram of Literature Search and Selection Criteria^(1)^

Figure 1 shows a flowchart describing the processes of identification, inclusion, and exclusion of the analysed articles. A total of 1395 articles were retrieved during selection phase 1. A total of 104 articles were selected in phase 2, of which 87 were excluded (see Supplementary Material 1). Therefore, 17 articles were included in the qualitative-quantitative analysis.

### Description of the studies

All studies were published in English in several countries in the last ten years.

All of the studies included were published between 2015 and 2020. Nine apps were found: *uHear* ^(22-25)^, *uHearing* Test^(25)^, *EarScale* ^(26)^, *HearScreen* ^(27,28)^, *HearingTest* ^(29,30)^, *Digits-in-Noise Test* ^(10,31-34)^, hearTest^(35,36)^, *Free Field Hearing* (FFH)^(37)^, *Free Hearing Test* ^(37)^. The most investigated apps were Digits-in noise Test (5 articles) and uHear (4 articles).

All apps found in this study used pure tone as the auditory stimulus^(23-30,35-37)^, except for *Digits-in-Noise Test* ^(10,31-34)^, which used speech stimulation through digits and Free field hearing (FFH)^(37)^ which used words.

### Risk of bias

The methodology of the selected studies was evaluated by using the Quality Assessment Tool for Diagnostic Precision Studies (QUADAS) with 14 items. Methodological limitations were identified in most of the included studies. One unclear articles in the 'patient selection' domain^(26)^, seven unclear articles in the 'index test' domain^(23,27-29,35-37)^, three unclear article in the 'reference standard' domain^(24,30,36)^, and two unclear articles in the 'flow and time' domain^(24,27)^. Regarding the applicability of studies, three articles obtained a high risk of bias in the 'patient selection' domain^(23,26,35)^ and one unclear article^(31)^. Five studies showed a low risk of bias for all domains according to QUADAS-2: Barczik and Serpanos^(25)^, Sousa et al.^(33,34)^, Potgieter et al.^(10)^ and Szudek et al.^(22)^.

**Figure 2:**
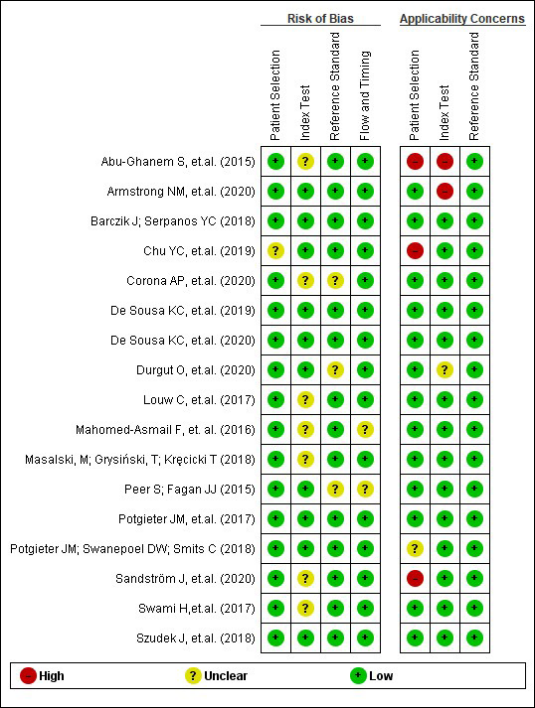
Quality assessment through the Quality Assessment Tool for Diagnostic Accuracy Studies-2 (QUADAS-2)

The results for the quality assessment are summarised in Figure 2.

### Synthesis of results

The authors of this study extracted the absolute values of the hearing screening test and pure-tone audiometry test from the studies to perform the calculations so that positive and negative predictive values, prevalence, and accuracy could be established. Some studies did not present the absolute values that would allow calculations, so their authors were contacted. However, such information could not be obtained on time from seven studies.

Invalid cut-off values were selected for sensitivity and specificity analysis, in which values >80% were considered excellent results, 70-80% good, 60-69% reasonable and <60% unfavorable results for a screening test^(38)^. The sensitivity of the selected studies varied substantially between good results (73% for the study by Swami et al.^(37)^), and excellent (100% in the studies by Abu-Ghanem et al.^(23)^, Peer and Fagan^(24)^, and Corona et al.^(36)^), while specificity varied between reasonable results (60% in the study by Abu-Ghanem et al.^(23)^) and excellent results (100% in studies by Corona et al.^(36)^ and Chu et al.^(26)^). An exception was found in the study by Barczik & Serpanos^(25)^, which performed a precision analysis according to three types of earphones and where a significant variability in the sensitivity and specificity values can be observed (see Table 1).

**Table 1:** Summary of studies included in the systematic review (n=17)

**Authors/ Year/Country**	**Sample/Age group (mean ± SD)**	**App**	**Type of Stimulus**	**Gold standard test**	**Gold standard test pass-fail criteria**	**Sensitivity**	**Specificity**	**PPV/NPV**	**Prevalence/Accuracy**
Abu-Ghanem, et al. (2015)^(23)^ Israel	26 individuals/ 65-94 years old (84.4 ± 6.73)	uHear	Pure tone	Pure-tone threshold audiometry from 250 to 6000 Hz	Mean of 500, 1000, and 2000 Hz at 40 dB as the cut-off point.	100%	60%	33.3% 100%	4.1% 91.6%
Armstrong et al. (2020)^(32)^ Netherlands	3422 individuals/ 51-98 years old	*Digits-in-Noise* (DIN) test	Digits-in-noise	Pure-tone threshold audiometry from 250 to 8000 Hz.	Mean of 500, 1000, 2000 and 4000 Hz at 25 dB as the cut-off point.	For mild hearing loss = 69% For moderate hearing loss = 94%	For mild hearing loss = 75% For moderate hearing loss = 95%	*	*
Barczik and Serpanos (2018)^(25)^ United States	22 individuals/ 18-84 years old (48.7)	*uHear* *uHearing Test*	Pure tone Pure tone	Pure-tone threshold audiometry from 250 to 8000 Hz	Mean of 500, 1000, and 2000 Hz at 25 dB as the cut-off point.	*UHear* *Earbud earphones =* 90-100% Supra-aural headphones=100% Circumaural headphones = 88,9-100% *uHearing Test* *Earbud earphones =* 10,5-75% Supra-aural headphones=85,7-90,5% Circumaural headphones = 10,5-70%	*uHear* *Earbud earphones =* 85,5-100% Supra-aural headphones=6,5-25% Circumaural headphones = 33-96,3% *uHearing Test* *Earbud earphones =* 91,7-100% Supra-aural headphones=87-86,7% Circumaural headphones = 100%	*uHear* *Earbud earphones =* 81,3-100%/ 92,3-100% Supra-aural headphones=31-52,6% Circumaural headphones = 55,6-86,7% *uHearing Test* *Earbud earphones =* 88,2-100%/ 59,5-81,5% Supra-aural headphones=90,9-93,6% Circumaural headphones = 100%/ 59,5-93,3%	*
Chu et al. (2019)^(26)^ Taiwan	85 individuals/ 11-12 years old (11 ±0,5)	*EarScale app*	Pure tone	Pure-tone threshold audiometry from 500 to 4000Hz	Mean of 500, 1000, 2000 Hz and 4000 Hz more than 25 dB in the sound-treated booth were designated as hearing impairment.	95,2-100%	100%	*	*
Corona et al. (2020)^(36)^ Brazil	300 adults/ 15-92 years old (53±*) 40 children/ 5-14 years old (9±*)	*hearTest*	Pure tone	Pure-tone threshold audiometry from 250 to 8000 Hz	Mean of 500, 1000, 2000 and 4000 Hz at 25 dB as the cut-off point.	Adults = 98% Children = 100%	Adults = 100% Children = 98%	Adults = 86,7% 99,2% Children = 83,3% 100%	*
Durgut et al. (2020)^(30)^ Turkey	50 individuals/ 5-15 years old (8.18±2.58)	*HearingTest*	Pure tone	Pure-tone threshold audiometry from 500 to 4000 Hz	Mean of 500, 1000, and 2000 Hz at 20 dB as the cut-off point.	93,6%	26,4%	53% 33%	47% 58%
Louw et al. (2017)^(28)^ South Africa	1236 individuals Above 16 years olds (37.8±17.9)	*HearScreen*	Pure tone	Pure-tone threshold audiometry from 250 to 8000 Hz	Mean of 500, 1000, 2000 Hz and 4000 Hz was greater than 25 dB for children or greater than 35 dB for adults.	81.7%	83.1%	86.6% 99.4%	*
Mahomed-Asmail et al. (2016)^(27)^ South Africa	1070 individuals/ 5–12 years old (8 ± 1.1)	*HearScreen*	Pure tone	Pure-tone threshold audiometry from 250 to 8000 Hz	Gold standard test results revealed hearing loss when a threshold is higher than 25 dB HL at 0.5, 1, 2, or 4 kHz.	75%	98.5%	52.9% 99.4%	*
Masalski et al. (2018)^(29)^ Poland	70 individuals/ 18–71 years old (36±11)	*HearingTest*	Pure tone	Pure-tone threshold audiometry from 250 to 8000 Hz	Hearing loss was diagnosed when the threshold exceeded 30 dB at one of the following frequencies: 500Hz, 1 kHz, 2 kHz, or 25 dB at more than one, or when the hearing threshold exceeded 50 dB at 4 kHz.	98%	79%	*	*
Peer and Fagan (2015)^(24)^ South Africa	25 individuals/ 15–80 years old (*)	*uHear*	Pure tone	Pure-tone threshold audiometry from 250 to 6000Hz	For the auditory screening, presence or absence of moderate or worse hearing loss (pure-tone average >40 dB) in each ear was determined via formal audiometry, considering 40 dB as the critical auditory threshold for disabling hearing loss.	100%	Waiting room = 64% Quiet room = 74% Acoustically treated room = 88%	100% in all environments Waiting room = 32% Quiet room = 72.7% Acoustically treated room = 100%	84% Waiting room = 50% Quiet room = 78% Acoustically treated room = 90%
Potgieter et al. (2018)^(10)^ South Africa	458 individuals/ 16-90 years old (27±16)	*Digits-in-Noise* (DIN) test	Digits-in-noise	Pure-tone threshold audiometry from 250 to 8000 Hz.	Mean of 500, 1000, 2000 and 4000 Hz at 25 dB as the cut-off point.	94%	77%	*	*
Potgieter et al. (2018)^(31)^ South Africa	109 individuals/ 16–89 years old (72±7.2)	*Digits-in-Noise* (DIN) test	Digits-in-noise	Pure-tone threshold audiometry from 250 to 8000 Hz	Mean of 500, 1000, 2000 and 4000 Hz at 25 dB as the cut-off point.	88%	88%	98.3% 75.5%	*
Sandström et al. (2020)^(35)^ South Africa	63 individuals/ 20-88 years old (52±*)	*hearTest*	Pure tone	Pure-tone threshold audiometry from 250 to 8000 Hz	Mean of 500, 1000, 2000 and 4000 Hz at 20 dB as the cut-off point.	90,6%	94,2%	*	38,1% *
Sousa et al. (2019)^(33)^ South Africa	145 individuals/ 18-84 years old (*)	*Digits-in-Noise (DIN) test*	Digits-in-noise	Pure-tone threshold audiometry from 250 to 8000 Hz.	Mean of 500, 1000, 2000 and 4000 Hz at 25 dB as the cut-off point.	Antiphasic DIN = 85% Diotic DIN = 83%	Antiphasic DIN = 67% Diotic DIN = 80%	*	*
Sousa et al. (2020)^(34)^ South Africa	158 individuals/ 18-92 years old (61±17)	*Digits-in-Noise* (DIN) test	Digits-in-noise	Pure-tone threshold audiometry from 250 to 8000 Hz.	Mean of 500, 1000, 2000 and 4000 Hz at 25 dB as the cut-off point.	97,2%	93,4%	*	*
Swami et al. (2017)^(37)^ India	200 individuals/ 17-65 years old (*)	*Free field hearing* (FFH) *Free Hearing Test* (“freehearingtestsoftware.com”)	Words Pure tone	Pure-tone threshold audiometry from 250 to 8000 Hz	Mean air conduction thresholds at a 500 Hz, 1000 Hz, 2000 Hz, 3000 Hz higher than 20 dB were considered normal.	FFH: 73% *Free Hearing Test*: 98%	FFH: 99% *Free Hearing Test*: 95%	Free Hearing Test = 95.1% Free Hearing Test = 97.9%	50% 96.5%
Szudek et al. (2012)^(22)^ Canada	100 individuals/ 20-91 years old (46±*)	*uHear*	Pure tone	Pure-tone threshold audiometry from 250 to 6000Hz	Mean of 500, 1000, 2000 and 4000 Hz at 40 dB as the cut-off point.	100%	90%	*	*

Authors did not provide the values in the manuscript or did not forward absolute values so the researchers of this review could perform calculations

**Caption:** VPP: positive predictive value; VPN: negative predictive value

### Additional analysis

The authors of this review calculated positive and negative predictive values, accuracy, and prevalence based on the absolute values made available in the analysed studies or sent by the researchers who were e-mailed. The calculated results are shown in Table 1.

## DISCUSSION

The applications Digits-in-noise Test (5 articles)^(10,31-34)^, uHear (4 articles)^(22-25)^, HearScreen (2 articles)^(27,28)^, hearTest (2 articles)^(35,36)^ and Hearing Test (2 articles)^(29,30)^ were the most studied. The others applications were EarScale, uHearing Test, Free field hearing (FFH) and Free Hearing Test.

In general, it is consistent across all studies included in this systematic review that using quick and accessible methods for hearing screening is necessary for the current hearing healthcare situation. Hearing screening is a common approach used by professionals to raise public awareness and promote intervention^(39)^.

App-based hearing screening is accessible to the public, so it has become an instrument for health promotion. Studies suggest that it improves patients' adherence to treatment because it offers information on their condition, which allows them to question their treatment. Besides, they can store their results and check previous screenings, as well as access healthcare services for diagnosis^(40,41)^.

Based on that information and the results of this review, it is clear that the uHear app has been used as a research tool on this subject and has shown effectiveness for identifying more significant hearing losses since individuals with more considerable hearing losses tend to fail this screening. Szudek et al.^(22)^ applied such screening methods to individuals older than 18 years and concluded it was a reasonable test to dismiss moderate hearing loss.

Abu-Ghanem et al. ^(23)^ used the uHear app with an elderly population and noticed that this screening method showed excellent sensibility (100%) and fair specificity (60%). Also, it proved to be a practical and useful screening tool for hearing loss in the elderly population since it is free to download and easy to use. Furthermore, this method is well accepted by the elderly and is also useful in dismissing significant hearing loss. However, the study emphasises the need for further research to determine an ideal cut-off point before it can be routinely used as a screening tool for geriatric oncology purposes.

UHear was also studied by Barczik and Serpanos^(25)^ along with the uHearingTest application, where the accuracy was verified according to the type of headset (earbud earphones, supra-aural headphones, and circumaural headphones) and with the different pure tone frequencies. The authors concluded that earbuds showed better sensitivity and specificity values for uHear, and the supra-aural headphone proved to be more accurate for uHearingTest. The researchers emphasize how important it is to use applications in hearing screening exclusively with the appropriate transducers.

On the other hand, Peer and Fagan^(24)^ used uHear in a small population with an extensive age range, including adult and elderly patients, and applied the test in different environments. They concluded that the sensitivity of the uHear app for iPhone is excellent (100% sensitive) to track disabling hearing loss and has better accuracy for high-frequency hearing loss in silent or acoustically-treated rooms than in waiting rooms, but showed variable specificity values (64–88%) according to the environment tested.

HearScreen is another app that has proven to be accurate. It has been used with a younger, school-aged population and is considered a cheap alternative to conventional audiometry without significant differences between the results of hearing screening tests and conventional audiometry^(42)^. Louw et al.^(28)^ used the HearScreen app in primary care clinics in 1,236 individuals older than 16 years and concluded that the method is effective and can become a tool for early identification of hearing loss. Mahomed-Asmail et al.^(27)^ used the same app on more than 1,000 school-aged children and also concluded that it delivers a low-cost, accurate, and efficient screening solution at school.

EarScale was yet another application used for hearing screening of 85 students. The app proved to be an accurate method to identify more significant hearing losses in that population, with sensitivity values between 95.2-100% and specificity of 100^(26)^.

Masalski et al.^(29)^ used the Hearing Test screening tool in their study. It showed excellent sensitivity (98%) and good specificity (79%), confirming the potential application in hearing monitoring, screening tests, or epidemiological tests on a large scale. Durgut et al.^(30)^ used the Hearing Test compared to conventional audiometry to assess hearing thresholds in children with Otitis Media with Effusion (OME) and to determine the accuracy and reliability of this method. They concluded that there was no statistically significant correlation between the screening result by the application with the average of pure tone thresholds of conventional audiometry, since it presented a very low specificity value (26.4%) indicating that it is not an appropriate screening test for to detect hearing loss in children with OME.

The HearTest is based on the validated hearing screen technology. Sandström et al.^(35)^ studied this application, obtained high specificity and sensitivity values ​​(94.2% and 90.6%, respectively), concluding that it is an effective method for identifying hearing losses. However, a limitation related to differences in responses was discussed when the test is performed by the individual (self-test) and when there is a facilitator during its performance. Thinking about it, Corona et al.^(36)^ also investigated hearTest in these conditions and with a sample composed of children and adults and observed that sensitivity and specificity were> 90% to identify disabling hearing loss for both response modes (self-test) or facilitator with adults and children. They also found a similar sensitivity value in identifying any level of hearing loss for both response modes in children, with specificity> 80%, and for the self-test mode in adults. Low specificity was observed when identifying any level of hearing loss in adults using the facilitator test.

In this systematic review, 11 of 17 included articles used pure tone stimuli. Only five articles used digits in noise, while one article used both pure tone and words as their stimuli on the smartphone.

The study by Swami et al.^(37)^ compared two hearing screening methods: The Free Field Hearing (FFH) program, which used speech stimulus (disyllabic list); and the Free Hearing Test app, which used pure tone. This study was the only that used speech stimulus (words). The Free Hearing Test app was more efficient than the FFH, and it can be particularly useful in places where pure-tone audiometry facilities are not available. The authors highlighted that speech stimuli are better to measure individuals' actual communicative function abilities^(43-46)^. However, the use of words may have been influenced by factors such as the subjects' language skills, language fluency, or social and environmental aspects, for example.

Smits et al.^(15)^ state that it is essential to use familiar words in a closed set rather than open sentences to reduce the effects of pre-existing language skills difficulties on the test result. A prevalent category is digits as they belong to the most spoken words in any age group.

The study by Potgieter et al.^(31)^ used an app involving hearing screening through speech material (digits) with background noise called *Digits-in-Noise Test*. The app is essential to provide additional information on the impairment of speech recognition in noise and also has excellent sensitivity (88%) and specificity (88%) to identify auditory alterations. Using digits as a stimulus has been widely accepted by researchers since language fluency or social and environmental factors do not influence the output.

Potgieter et al.^(10)^ also verified the accuracy of Digits-in-Noise Test and the influence of factors such as age, the degree of hearing loss, and linguistic competence between South African English native and non-native speakers. They observed age and linguistic competence had a significant impact in identifying individuals with hearing loss and that it is an accurate method, with 94% sensitivity and 77% specificity. It is worth nothing that, although digits reduce the effects of language skills, there is still an influence of language on the test result, which requires further studies.

Souza et al.^(33)^ studied the use of the Digit-in-Noise Test using digits that are phase inverted (antiphasic) between the ears, while leaving the masking noise interaurally in-phase. Such a configuration of stimuli was shown to improve of the Digit-In-Noise Test SRTs in normal hearing listeners^(47)^. They started from the hypothesis that homophasic diotic or monoaural stimuli may not be sensitive to detect unilateral, asymmetric or conductive hearing losses. They concluded with this study that the use of this test with antiphasic stimuli proved to be more sensitive (85%) to detect unilateral and asymmetric and conductive hearing losses in relation to the presentation of homophasic diotic stimuli (83%), however it proved to be less specific in this case. correlation (60%).

Still on conductive hearing losses, Sousa et al.^(34)^ investigated whether the use of DIN Test is effective to detect this type of hearing loss and concluded that the use of the application combined with the research of pure tone thresholds by air has good precision and high sensitivity and specificity (97.2% and 93.4%, respectively).

There are no standard cutoff points for the DIN test. These cutoff points tend to vary according to the study. Koole et al.^(48)^ reported that an appropriate cutoff point for the DIN test to identify abnormal hearing would generally be in the range between 0 and signal-to-noise ratio of 5 decibels (dB SNR). Dawes et al.^(49)^ used the following cutoff points: <-5.5dB SNR for normal hearing performance, -5.5dB SNR to -3.5dB SNR for insufficient hearing performance, and> SNR of -3.5dB for auditory performance bad.

Armstrong et al.^(32)^ sought to establish cutoff points for the DIN test according to age and sex through a population-based study and obtained the following categories: SNR <-5.55dB (normal), between -5.55 and - 3.80dB SNR (insufficient) and> -3.80dB SNR (poor). They did not find differences between age, sex and / or age and sex and that the DIN test showed high values ​​of sensitivity and specificity to detect moderate hearing losses (94% and 95%, respectively), agreeing with most studies that reveal their importance in identifying more significant hearing losses.

In general, all studies included in this review provided important information to clarify which applications can be considered accurate for the identification of hearing loss. However, bias analysis showed that there is a need for more accurate information, such as describing the procedures for applying the tests and the reference standard, as well as a more substantial concern that such methods are replicated in other research.

Based on all the results of this systematic review, all studies point to the need to test the application so that early detection of hearing loss is increasingly accessible to all changes, whether in urban or rural areas and all age groups.

This systematic review investigated the accuracy of smartphone apps to identify hearing loss. One of the limitations of this study was the exclusion of many articles because their methodologies did not contain enough data to support precise values of accuracy, prevalence and predictive values. Another limitation was the lack of ability to perform a meta-analysis due to the heterogeneity of analysis requests between the index tests (applications) and the reference test (pure tone audiometry). In this regard, it was observed that some studies used the mean threshold reference test at 20, or 25 or 40 dB regardless of the age range of the investigated subjects. This difference in the criteria of the reference test can affect the outcomes related to accuracy, therefore it is a limitation of the study that made the meta-analysis unfeasible. New studies involving the accuracy of these applications on a large scale and in all age groups need to be carried out.

## CONCLUSION

uHear, Digit-in-Noise Test, HearTest and HearScreen have shown significant values of sensitivity and specificity and can be considered as the most accurate methods for screening of hearing impairment.

## Appendix 1 Database search strategies

**Table:**

**Database**	**Search** (JULY 10^th^ 2018 updated on July 20^th^, 2020)
**LILACS**	audiology OR audiometria OR “audiometria de fala” OR “audiometria de tonos puros” OR “audiometria de habla” [Palavras] and smartphone OR teléfono inteligente OR telefone [Palavras] and Hearing loss OR Pérdida Auditiva OR perda auditiva [Palavras]
**PubMed**	(((“audiology” OR “audiometry” OR “speech audiometries” OR “speech audiometry” OR “pure-tone audiometry” OR “speech intelligibility” OR “speech intelligibilities” OR “speech reception threshold test” OR “speech discrimination tests” OR “speech discrimination test” OR “hearing test” OR “hearing tests” OR “screening test” OR “screening tests”)) AND (“hearing loss” OR “hearing impairment” OR hypoacusis OR hypoacuses OR “hearing disorder” OR “hearing disorders”)) AND (“cellular telephones” OR “mobile phone” OR “mobiles phones” OR “mobile telephone” OR mobile telephones” OR “mobile application” OR “mobile applications” OR “mobile app”)
**Scopus**	(TITLE-ABS-KEY (audiology OR audiometry) AND TITLE-ABS-KEY (“hearing loss” OR “hearing impairment”) AND TITLE-ABS-KEY (“mobile application” OR “mobile applications”))
**Web of Science**	**(**audiology OR audiometry OR “speech audiometries” OR “speech audiometry” OR “speech intelligibility” OR “speech intelligibilities” OR “speech discrimination tests” OR “speech discrimination test” OR “hearing test” OR “hearing tests” OR “screening test” OR “screening tests”) AND TÓPICO: (“hearing loss” OR “hearing impairment” OR hypoacusis OR hypoacuses OR “hearing disorder” OR “hearing disorders”) AND TÓPICO: (“cellular telephones” OR “mobile phone” OR “mobiles phones” OR “mobile application” OR “mobile applications” OR “mobile app”)
**Google Scholar**	audiometry AND hearing loss AND smartphone
**Open Grey**	hearing loss AND application
**ProQuest**	noft(audiology OR audiometry OR “speech audiometry” AND “hearing loss” OR “hearing impairment”) AND noft(“mobile application” OR “mobile applications” OR smartphone)

## Supplementary Material

Supplementary material accompanies this paper.

Supplementary Material 1 Excluded articles and reason for exclusion (n=87).

This material is available as part of the online article from https://www.scielo.br/j/CODAS
