# Supplementary material for: Accuracy of smartphone-based hearing screening tests: a systematic review
Source: Codas. 2022 Feb 23;34(3):e20200380. doi: 10.1590/2317-1782/20212020380 (PMC9769434; doi:10.1590/2317-1782/20212020380)
Supplement: Supplementary Material 1 [file codas-34-3-e20200380-suppl1.pdf]

Supplementary Material 1 – Excluded articles and reason for exclusion (n=87).

Author, Year	Reason for exclusion
Amlani[1]	4
Arthi and Sreenivas [2]	3
Azarov et al.[3]	4
Bauer et al.[4]	1
Bornman et al.[5]	4
Brennan-Jones et al.[6]	1
Bright et al.[7]	3
Brook and Williams[8]	5
Byrne, Schmitt and Murphy[9]	5
Chauhan and Shah[10]	4
Chen and Wang[11]	4
Chen et al.[12]	4
Choi et al.[13]	3
Colsman et al.[14]	1
Corrêa, Soldera and Machado[15]	5
Costa, Eto & Lucas[16]	1
Derin et al.[17]	4
Dewyer et al.[18]	4
DiGiovanni and Rizzo[19]	4
Dritsakis et al.[20]	4
Fetscher[21]	3
Ganesan, et al.[22]	3
Garrison e Bochner[23]	1
Garrison et al.[24]	1
Han et al.[25]	4
Hong, et al.[26]	2
Hollander et al.[27]	3
Hussein et al.[28]	3
Jacobs and Saunders[29]	3
Jacobs et al.[30]	5
Jayawardena et al.[31]	3
Kam et.al.[32]	4
Kam and Fu[33]	1
Kelly et al.[34]	1
Krzyzek et al.[35]	4
Laitinen et al.[36]	6
Larrosa et.al.[37]	4
Leonidas et al.[38]	3
Liu et al.[39]	1
Livshitz et al.[40]	1
Löhler et al.[41]	1
Lycke et al.[42]	4
Lycke et al.[43]	1
Magro et al.[44]	1
Mahomed-Asmail[45]	6

Martinez-Beneyto et al.[46]	4
Masalski et al.[47]	3
Messmer et al.[48]	6
Monica et al.[49]	1
Na et al.[50]	4
Nakamura[51]	3
Ogah[52]	4
Oliveira et al.[53]	6
Ondáš et al.[54]	1
Ozdek et al.[55]	1
Paglialonga et al.[56]	3
Paglialonga, Tognola and Pinciroli[57]	3
Paglialonga, Tognola and Pinciroli[58]	5
Paz-Oliveira et al.[59]	3
Pereira et al.[60]	4
Pickens et al.[61]	4
Potgieter et al. [62]	3
Rashid, Dreschler and Laat[63]	1
Saibua, Seesutas and Iransena[64]	3
Saliba et al. [65]	1
Saly[66]	4
Samelli et al. [67]	1
Samelli et al. [68]	1
Schönborn et al. [69]	4
Sethi et al.[70]	5
Sheikh and Sheikh[71]	1
Skarżyński et al.[72]	1
Sohn et al.[73]	4
Souza et al.[74]	1
Teixeira et al.[75]	6
Teki, Kumar and Griffiths[76]	3
Tognola et al.[77]	3
Tonder et. al.[78]	4
Vijayasingam et al.[79]	1
Vlaming et al.[80]	4
Wongsirichot et al.[81]	4
Xing et.al.[82]	5
Yang et al.[83]	5
Yao et al.[84]	1
Yeung et al.[85]	1
Yimtae et al.[86]	1
Zarei[87]	6

Legend: 1. Studies that did not use phone apps; 2. Studies that were not audiology-related; 3. Studies that did not compare smartphone app-based diagnostic methods with the reference standard (audiometry); 4 Studies that did not show any validity measurements (sensitivity and specificity) or did not show sufficient data to calculate them; 5. Comments, letters, conference, summary, personal opinions, and clinical trials; 6. Unavailable studies.

REFERENCES

1. Amlani AM. Improving Patient Compliance to Hearing Healthcare Services and Treatment through Self-Efficacy and Smartphone Applications. 2015. p. 16-.

2. Arthi S, Sreenivas TV, editors. Binaural spatial audiometry screening using android mobile device audio I/O facility2020/02//: Institute of Electrical and Electronics Engineers Inc.

3. Azarov E, Vashkevich M, Petrovsky N, Likhachov D, Petrovsky A. Petralex: A multiplatform hearing aid application for real-life communications and listening to digital audio. submitted to E-health. 2015.

4. Bauer MA, Sales A, Teixeira AR, Morsch P, Lessa AH, Bós ÂJG. Development and accuracy of a hearing screening application. Brazilian Journal of Otorhinolaryngology. 2020.

5. Bornman ME. Validation of hearTest Smartphone Application for Extended High Frequency Hearing Thresholds 2017.

6. Brennan-Jones CG, Eikelboom RH, Bennett RJ, Tao KFM, Swanepoel DW. Asynchronous interpretation of manual and automated audiometry: Agreement and reliability. Journal of Telemedicine and Telecare. 2018;24(1):37-43.

7. Bright T, Mulwafu W, Phiri M, Jiang F, Swanepoel DW, Kuper H, et al. Field test of the Rapid Assessment of Hearing Loss survey protocol in Ntcheu district, Malawi. International Journal of Audiology. 2020;59(8):574-82.

8. Brook LJ, Williams PAH. Developing a Mobile Audiometric Sound Booth Application for Apple IOS Devices. Australian eHealth Informatics and Security Conference. 2013.

9. Byrne DC, Schmitt L, Murphy WJ. Comparison of headphones for audiometric screening and hearing protector fit-testing. The Journal of the Acoustical Society of America. 2015;137(4):2293-.

10. Chauhan N, Shah J. Smart Phone Based Audiometry in City Traffic Police. Indian Journal of Otolaryngology and Head and Neck Surgery. 2018;70(3):342-5.

11. Chen F, Wang S, editors. Smartphone-based self hearing diagnosis using hearing aids: Institute of Electrical and Electronics Engineers (IEEE).

12. Chen F, Wang S, Li J, Tan H, Jia W, Wang Z. Smartphone-Based Hearing Self-Assessment System Using Hearing Aids with Fast Audiometry Method. IEEE Transactions on Biomedical Circuits and Systems. 2018;13(1):170-9.

13. Choi JM, Sohn J, Ku Y, Kim D, Lee J. Phoneme-based self hearing assessment on a smartphone. IEEE Journal of Biomedical and Health Informatics. 2013;17(3):526-9.

14. Colsman A, Supp GG, Neumann J, Schneider TR. Evaluation of Accuracy and Reliability of a Mobile Screening Audiometer in Normal Hearing Adults. Frontiers in Psychology. 2020;11:744-.

15. Corrêa A, Soldera C, Machado M. Audiometric Characterization of Adults and Elderly Patients Referred to a Hearing Evaluation. International Archives of Otorhinolaryngology. 2014;18(S 01):a2114-a.

16. Costa KMd, Eto J, Lucas PdA. Audiological profile of indoor cycling teachers. Revista CEFAC. 2020;22(2):14619-.

17. Derin S, Cam OH, Beydilli H, Acar E, Elicora SS, Sahan M. Initial assessment of hearing loss using a mobile application for audiological evaluation. Journal of Laryngology and Otology. 2016;130(3):248-51.

18. Dewyer NA, Jiradejvong P, Henderson Sabes J, Limb CJ. Automated smartphone audiometry: Validation of a word recognition test app. Laryngoscope. 2018;128(3):707-12.

19. DiGiovanni JD, Rizzo S. Wearable device for relieving tinnitus, hyperacusis and/or hearing loss. Google Patents; 2014.

20. Dritsakis G, Kikidis D, Koloutsou N, Murdin L, Bibas A, Ploumidou K, et al. Clinical validation of a public health policy-making platform for hearing loss (EVOTION): Protocol for a big data study. BMJ Open. 2018;8(2):20978-.

21. Fetscher E. The Clinical Relevance of Smartphone Applications in Medicine and Audiology. Communication Sciences and Disorders: Student Scholarship & Creative Works. 2016.

22. Ganesan P, Schmiedge J, Manchaiah V, Swapna S, Dhandayutham S, Kothandaraman PP. Ototoxicity: A challenge in diagnosis and treatment. Korean Audiological Society and Korean Otological Society; 2018. p. 59-68.

23. Garrison WM, Bochner JH. An Application for Screening Gradual-Onset Age-Related Hearing Loss. Health. 2017;09(04):715-26.

24. Garrison JR, Bond R, Gibbard E, Johnson MK, Simons JS. Monitoring what is real: The effects of modality and action on accuracy and type of reality monitoring error. Cortex. 2017;87:108-17.

25. Han JH, Yi DW, Lee J, Chang WD, Lee HJ. Development of a smartphone-based digits-in-noise test in korean: A hearing screening tool for speech perception in noise. Journal of Korean Medical Science. 2020;35(21).

26. Hong H, Dowdy DW, Dooley KE, Francis HW, Budhathoki C, Han HR, et al. Aminoglycoside-induced hearing loss among patients being treated for drug-resistant tuberculosis in South Africa: A prediction model. Clinical Infectious Diseases. 2020;70(5):917-24.

27. Hollander C, Joubert K, Schellack N. An ototoxicity grading system within a mobile app (OtoCalc) for a resource-limited setting to guide grading and management of drug-induced hearing loss in patients with drug-resistant tuberculosis: Prospective, cross-sectional case series. JMIR mHealth and uHealth. 2020;8(1):e14036-e.

28. Hussein SY, Swanepoel DW, Mahomed F, Biagio de Jager L. Community-based hearing screening for young children using an mHealth service-delivery model. Global Health Action. 2018;11(1):1467077-.

29. Jacobs PG, Saunders GH. New opportunities and challenges for teleaudiology within department of veterans affairs. Rehabilitation Research and Development Service; 2014. p. vii-xii.

30. Jacobs PG, Silaski G, Wilmington D, Gordon S, Helt W, McMillan G, et al. Development and evaluation of a portable audiometer for high-frequency screening of hearing loss from ototoxicity in homes/clinics. IEEE Transactions on Biomedical Engineering. 2012;59(12 PART2):3097-103.

31. Jayawardena ADL, Nassiri AM, Levy DA, Valeriani V, Kemph AJ, Kahue CN, et al. Community health worker-based hearing screening on a mobile platform: A scalable protocol piloted in Haiti. Laryngoscope Investigative Otolaryngology. 2020;5(2):305-12.

32. Kam ACS, Sung JKK, Lee T, Wong TKC, Van Hasselt A. Clinical evaluation of a computerized self-administered hearing test. International Journal of Audiology. 2012;51(8):606-10.

33. Kam ACS, Fu CHT. Screening for hearing loss in the Hong Kong Cantonese-speaking elderly using tablet-based pure-tone and word-in-noise test. International Journal of Audiology. 2019;59(4):301-9.

34. Kelly EA, Stadler ME, Nelson S, Runge CL, Friedland DR. Tablet-based Screening for Hearing Loss: Feasibility of Testing in Nonspecialty Locations. Otology and Neurotology. 2018;39(4):410-6.

35. Krzyżek K, Maciej K. Hearing tester - Mobile application for hearing loss screening. Diagnostyka. 2019;20(3):87-95.

36. Laitinen K. A comparison among hearing healthcare digital applications: An examination of test accuracy and user perception in normal hearing young adults 2016.

37. Larrosa F, Rama-Lopez J, Benitez J, Morales JM, Martinez A, Alañon MA, et al. Development and evaluation of an audiology app for iPhone/iPad mobile devices. Acta Oto-Laryngologica. 2015;135(11):1119-27.

38. Leonidas AVH, Paulina TPV, Rubén CCJ, Sebastián CAF. Methods used in mobile applications for the diagnosis of hearing loss: A systematic mapping study. KnE Engineering. 2020;2020:89-107-89-.

39. Liu Y, Yang D, Xiong F, Yu L, Ji F, Wang Q-J. Development and Validation of a Portable Hearing Self-Testing System Based on a Notebook Personal Computer. Journal of the American Academy of Audiology. 2015;26(08):716-23.

40. Livshitz L, Ghanayim R, Kraus C, Farah R, Even-Tov E, Avraham Y, et al. Application-Based Hearing Screening in the Elderly Population. Annals of Otology, Rhinology and Laryngology. 2017;126(1):36-41.

41. Löhler J, Lehmann M, Segler V, Volkenstein S, Battmer RD, Ernst A, et al. The sensitivity and specificity of the mini-audio-test (mat) for different levels of hearing loss. Laryngo- Rhino- Otologie. 2020;99(6):400-6.

42. Lycke M, Debruyne PR, Lefebvre T, Martens E, Ketelaars L, Pottel H, et al. The use of uHear™ to screen for hearing loss in older patients with cancer as part of a comprehensive geriatric assessment. Acta Clinica Belgica: International Journal of Clinical and Laboratory Medicine. 2018;73(2):132-8.

43. Lycke M, Lefebvre T, Cool L, Van Eygen K, Boterberg T, Schofield P, et al. Screening Methods for Age-Related Hearing Loss in Older Patients with Cancer: A Review of the Literature. Geriatrics. 2018;3(3):48-.

44. Magro I, Clavier O, Mojica K, Rieke C, Eisen E, Fried D, et al. Reliability of Tablet-based Hearing Testing in Nicaraguan Schoolchildren: A Detailed Analysis. Otology and Neurotology. 2020;41(3):299-307.

45. Mahomed-Asmail F. School-based hearing screening and diagnosis using automated and mobile health technologies: University of Pretoria; 2015.

46. Martinez-Beneyto P, Franchella S, Rodriguez FA, Navarro-Velasquez R, Martinez-Beneito MA, Martini A, et al. Are smartphone applications (App) useful to improve hearing? Acta Otorhinolaryngologica Italica. 2020;40(4):304-10.

47. Masalski M, Kipiński L, Grysiński T, Krêcicki T. Hearing tests on mobile devices: Evaluation of the reference sound level by means of biological calibration. Journal of Medical Internet Research. 2016;18(5):e130-e.

48. Messmer C, Zirn S, Schuster M. Central or combined central and peripheral hearing loss at an early born child. Neuropediatrics. 2013;44(02).

49. Monica SD, Ramkumar V, Krumm M, Raman N, Nagarajan R, Venkatesh L. School entry level tele-hearing screening in a town in South India – Lessons learnt. International Journal of Pediatric Otorhinolaryngology. 2017;92:130-5.

50. Na Y, Joo H, Yang H, Kang S, Hong S, Woo J. Smartphone-Based Hearing Screening in Noisy Environments. Sensors. 2014;14(6):10346-60.

51. Nakamura N, editor Development of mobile audiometric test system using mobile phones2003: Institute of Electrical and Electronics Engineers Inc.

52. Ogah SA. Hearing loss assessment: evaluating the uHear tm an ios-based application as a screening tool. Pakistan Journal of Medicine and Dentistry. 2017;6(01).

53. Oliveira P, Souza I, Pedrosa L, Santos R, Santos T, Carneiro T. Hearing Handicap Inventory for Adults and Audiological Findings in Cancer Patients. International Archives of Otorhinolaryngology. 2014;18(S 01):a2284-a.

54. Ondáš S, Kiktová E, Pleva M, Oravcová M, Hudák L, Juhár J, et al. Pediatric speech audiometry web application for hearing detection in the home environment. Electronics (Switzerland). 2020;9(6):1-15.

55. Ozdek A, Karacay M, Saylam G, Tatar E, Aygener N, Korkmaz MH. Comparison of pure tone audiometry and auditory steady-state responses in subjects with normal hearing and hearing loss. European Archives of Oto-Rhino-Laryngology. 2010;267(1):43-9.

56. Paglialonga A, Tognola G, Grandori F. A user-operated test of suprathreshold acuity in noise for adult hearing screening: The SUN (Speech Understanding in Noise) test. Computers in Biology and Medicine. 2014;52:66-72.

57. Paglialonga A, Tognola G, Pinciroli F, editors. Apps for Hearing Healthcare2015: IOS Press.

58. Paglialonga A, Tognola G, Pinciroli F. Apps for Hearing Science and Care. American Journal of Audiology. 2015;24(3):293-8.

59. Paz-Oliveira A, Momensohn-Santos TM, Carmo MPd, Fiore A. Testes de fala no ruído na clínica audiológica – Uma Revisão Integrativa. Distúrbios da Comunicação. 2020;32(1):124-39.

60. Pereira O, Pasko LE, Supinski J, Hammond M, Morlet T, Nagao K. Is there a clinical application for tablet-based automated audiometry in children? International Journal of Pediatric Otorhinolaryngology. 2018;110:87-92.

61. Pickens AW, Robertson LD, Smith ML, Zheng Q, Song S. Headphone evaluation for app-based automated mobile hearing screening. International Archives of Otorhinolaryngology. 2018;22(4):358-63.

62. Potgieter JM, Swanepoel DW, Myburgh HC, Hopper TC, Smits C. Development and validation of a smartphone-based digits-in-noise hearing test in South African English. International Journal of Audiology. 2016;55(7):405-11.

63. Rashid MS, Dreschler WA, Laat JAPM. Evaluation of an internet-based speech-in-noise screening test for school-age children. International Journal of Audiology. 2017;56(12):967-75.

64. Saibua S, Seesutas S, Israsena P, editors. Low-Noise High-Power Amplification System Design for Mobile Tablet-Based Audiometry2017; Midview City, SGP: Singapore Therapeutic, Assistive & Rehabilitative Technologies (START) Centre.

65. Saliba J, Al-Reefi M, Carriere JS, Verma N, Provencal C, Rappaport JM. Accuracy of Mobile-Based Audiometry in the Evaluation of Hearing Loss in Quiet and Noisy Environments. Otolaryngology - Head and Neck Surgery (United States). 2016;156(4):706-11.

66. Saly GL, inventorAudiometric Testing Devices and Methods2013.

67. Samelli AG, Rabelo CM, Sanches SGG, Aquino CP, Gonzaga D. Tablet-Based Hearing Screening Test. Telemedicine and e-Health. 2017;23(9):747-52.

68. Samelli AG, Rabelo CM, Sanches SGG, Martinho AC, Matas CG. Tablet-based tele-audiometry: Automated hearing screening for schoolchildren. Journal of Telemedicine and Telecare. 2018;26(3):140-9.

69. Schönborn D, Asmail FM, De Sousa KC, Laplante-Lévesque A, Moore DR, Smits C, et al. Characteristics and Help-Seeking Behavior of People Failing a Smart Device Self-Test for Hearing. American Journal of Audiology. 2020;29(3):365-74.

70. Sethi RKV, Ghanad I, Kanumuri V, Herrmann B, Kozin ED, Remenschneider AK. Mobile Hearing Testing Applications and the Diagnosis of Sudden Sensorineural Hearing Loss: A Cautionary Tale. Otology and Neurotology. 2018;39(1):e1-e4.

71. Sheikh W, Sheikh N. Audiometry: A model-view-viewmodel (MVVM) application framework for hearing impairment diagnosis. Journal of Open Source Software. 2020;5(51):2016-.

72. Skarzyński PH, Swierniak W, Gos E, Pierzyńska I, Walkowiak A, Cywka KB, et al. Results of hearing screening of school-age children in Bishkek, Kyrgyzstan. Primary Health Care Research and Development. 2020;21(e18):1-6.

73. Sohn J, Kim D, Ku Y, Lee K, Lee J, editors. Study on self hearing assessment using speech sounds2011.

74. Souza RRD, Vieira MG, Júnior CJFL. The integral child health care network in the federal district – Brazil. Ciencia e Saude Coletiva. 2019;24(6):2075-84.

75. Teixeira A, Bressanelli A, Gonçalves A, Benin L, Olchik M, Fraga R. Correlation between Hearing and Memory in Healthy Elderly. International Archives of Otorhinolaryngology. 2014;18(S 01):a2143-a.

76. Teki S, Kumar S, Griffiths TD. Large-Scale Analysis of Auditory Segregation Behavior Crowdsourced via a Smartphone App. PLOS ONE. 2016;11(4):e0153916-e.

77. Tognola G, Paglialonga A, Chiaramello E, Pinciroli F. eHealth for Hearing-New Views and Apps Practicalities. 2015.

78. Tonder JV, Swanepoel DW, Mahomed-Asmail F, Myburgh H, Eikelboom RH. Automated smartphone threshold audiometry: Validity and time efficiency. Journal of the American Academy of Audiology. 2017;28(3):200-8.

79. Vijayasingam A, Frost E, Wilkins J, Gillen L, Premachandra P, McLaren K, et al. Tablet and web-based audiometry to screen for hearing loss in adults with cystic fibrosis. Thorax. 2020;75(8):632-9.

80. Vlaming MSMG, Kollmeier B, Dreschler WA, Martin R, Wouters J, Grover B, et al. Hearcom: Hearing in the communication society. 2011. p. 175-92.

81. Wongsirichot T, Elz N, Suwanmanee N, Hmad-a-dam H, Plansangket S, Sukpisit S, editors. Hearing Performance Testing Application2017; New York, NY, USA: Association for Computing Machinery.

82. Xing Y, Fu Z, Wu X, Chen J, editors. Evaluation of Apple iOS-based automated audiometry2016.

83. Yang H, Pang J, Xiong H, Sun Y, Lai L, Chen S, et al. The protective effect of autophagy on ischemia/reperfusion-induced hearing loss. NeuroReport. 2017;28(17):1157-63.

84. Yao J, Yao D, Givens G. A Browser-Server-Based Tele-audiology System That Supports Multiple Hearing Test Modalities. Telemedicine and e-Health. 2015;21(9):697-704.

85. Yeung J, Javidnia H, Heley S, Beauregard Y, Champagne S, Bromwich M. The new age of play audiometry: Prospective validation testing of an iPad-based play audiometer. Journal of Otolaryngology - Head and Neck Surgery. 2013;42(MAR):21-.

86. Yimtae K, Israsena P, Thanawirattananit P, Seesutas S, Saibua S, Kasemsiri P, et al. A Tablet-Based Mobile Hearing Screening System for Preschoolers: Design and Validation Study. JMIR mHealth and uHealth. 2018;6(10):e186-e.

87. Zarei K. Objective quantification of sensory function using a battery of smartphone applications: University of Iowa; 2017.
